# Human rabies encephalomyelitis in the background of rabies outbreak in animals in Gelephu, Bhutan, 2023: a case report

**DOI:** 10.1186/s40249-023-01148-2

**Published:** 2023-10-16

**Authors:** Thinley Dorji, Jeewanath Lamichaney, Choeda Gyaltshen, Lungten Lungten, Guru Prasad Dhakal, Sithar Dorjee, Mimi Lhamu Mynak

**Affiliations:** 1 Department of Internal Medicine, Central Regional Referral Hospital, Gelephu, Bhutan; 2 Regional Veterinary Hospital and Epidemiology Centre, Gelephu, Bhutan; 3 Faculty of Postgraduate Medicine, Khesar Gyalpo University of Medical Sciences of Bhutan, Thimphu, Bhutan; 4 Office of the President, Khesar Gyalpo University of Medical Sciences of Bhutan, Thimphu, Bhutan; 5 Office of the President, National Medical Services, Ministry of Health, Thimphu, Bhutan

**Keywords:** Rabies, Outbreak, Encephalitis, Lyssavirus, Myelitis, Neglected diseases, One Health, Tropical medicine

## Abstract

**Background:**

Rabies continues to pose significant public health challenges in many developing countries including Bhutan. A probable case of rabies was admitted to our hospital and its reporting led to the uncovering of an outbreak in domestic and wild animals. We discuss the challenges in the diagnosis and management of rabies in a resource-limited setting.

**Case presentation:**

A 35-year-old male presented with intermittent fever, bilateral lower limb weakness that was rapidly progressive, urinary incontinence with episodes of palpitations and sweating. He had sustained a Category III bite on the right lower thigh with four bite marks, inflicted by a stray dog. He had received post-exposure prophylaxis with intra-dermal anti-rabies vaccine. On initial examination, the patient was in distress but cooperative for the interview. He had pulse rate ranging from 60 to 100/min with episodes of diaphoresis and palpitations, but with normal capillary blood glucose. In the lower limb, the muscle power was zero with absent tendon reflexes in the lower limb and impaired abdominal reflex below T_10_ level. He had hyperaesthesia below T_8_, hydrophobia, aerophobia and photophobia. He had multiple spontaneous fasciculations in both the thighs and right deltoid and these later involved the intercostal muscles, neck and face muscles. He had altered sensorium and desaturation for which he required mechanical ventilation. Polymerase chain reaction for rabies virus was negative in cerebrospinal fluid and saliva. Rabies virus neutralizing antibody was negative in cerebrospinal fluid but had high titres in the serum. He received Human Rabies Immunoglobulin after admission. He was managed in the intensive care unit and died 23 days later. After this case was notified, a rapid response team was deployed in the field, and uncovered rabies outbreak in animals in the locality.

**Conclusions:**

This case called for a serious evaluation of the country’s efforts in achieving zero rabies deaths by 2030. The management of this case identified several critical areas of context-specific interventions in Bhutan. There is also an urgent need to improve diagnostic capabilities at the national reference laboratory and enhance the technical competencies of healthcare workers in the management of dog bite cases.

**Graphical Abstract:**

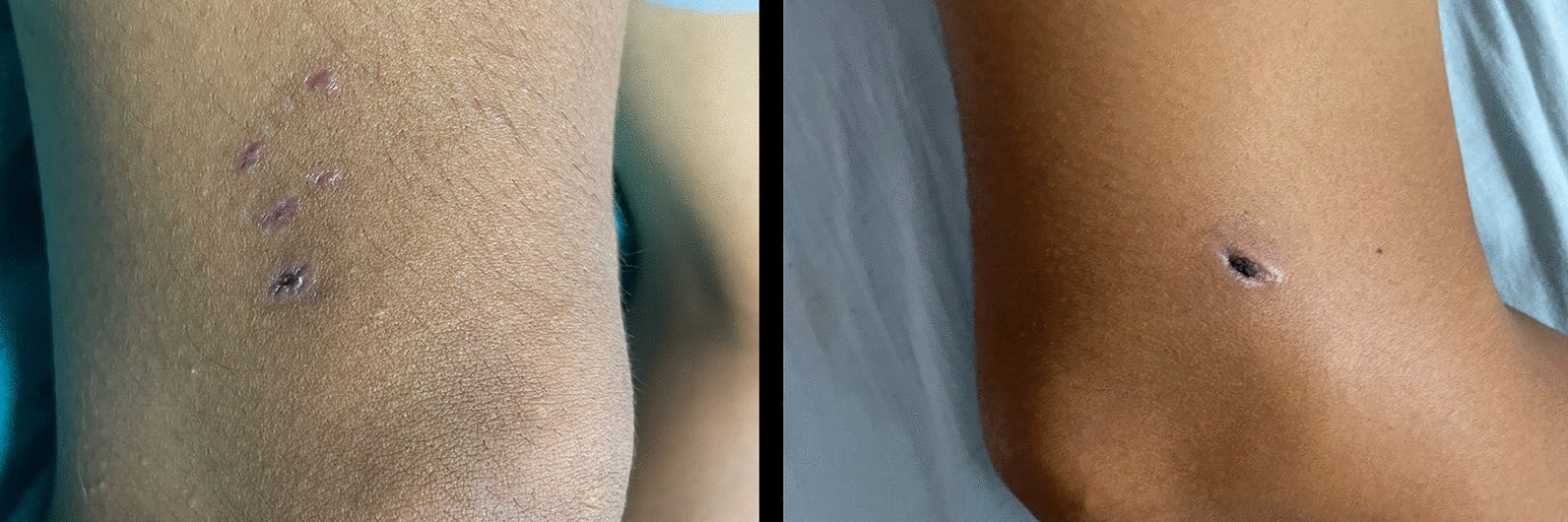

## Background

Rabies is one of the neglected tropical diseases that continues to pose significant public health concern in many developing countries [1]. Globally, the number of humans with rabies has decreased from 24,744 in 1990 to 14,075 in 2019 [2]. The South Asia region continues to report the highest proportion of all cases of rabies reported globally with the highest number of deaths occurring in India [2].

Human rabies results from an infection with rabies virus (RABV) that is transmitted in the majority of cases from infected dogs [1, 3]. Rabies virus infection in humans results in a furious or dumb forms that has four clinical stages: incubation, prodrome, acute neurological signs of encephalomyelitis and death [3]. Post-exposure prophylaxis with anti-rabies vaccine administered as per World Health Organization (WHO) categories of animal bite wounds is highly effective in preventing rabies infection. However, breakthrough rabies infections have resulted in those who have received post-exposure prophylaxis [4]. Victims sustaining multiple wounds and bites to the head, neck and face and poor practices related to post-exposure prophylaxis have been cited as common reasons [4].

Bhutan is a small country in the eastern Himalayas with its southern border adjoining the north-eastern parts of India. Bhutan has a high stray dog population, estimated at 72,000 in 2021. Dog bites are a common problem with 7083 dog bites reported in 2019 and 6873 in 2021 [5]. The country implemented the Nationwide Accelerated Dog Population Management programme since 2021 that includes neutering and vaccinating the dogs [6]. In June–July 2023, Bhutan reported its 19th human rabies case that led to the uncovering of an outbreak of rabies in domestic (dogs, cats, cattle) and wild (rabbit) animals in Gelephu across seven sub-districts. We report the details of rabies in humans and the challenges in diagnosing and managing such cases in a resource-limited setting.

## Case presentation

### History and examination

A 35-year-old male presented to our hospital in June 2023 with intermittent fever for 4 days, and bilateral lower limb weakness for 3 days that was sub-acute in onset, progressive with patient becoming bed-bound within 1 day. He had sensation of bladder filling but was unable to void urine. He also had episodes of palpitations and excessive sweating and nausea. There was no tightness or looseness of limbs, no cough, chest pain, abdominal pain, seizures or loss of consciousness.

**Fig. 1:**
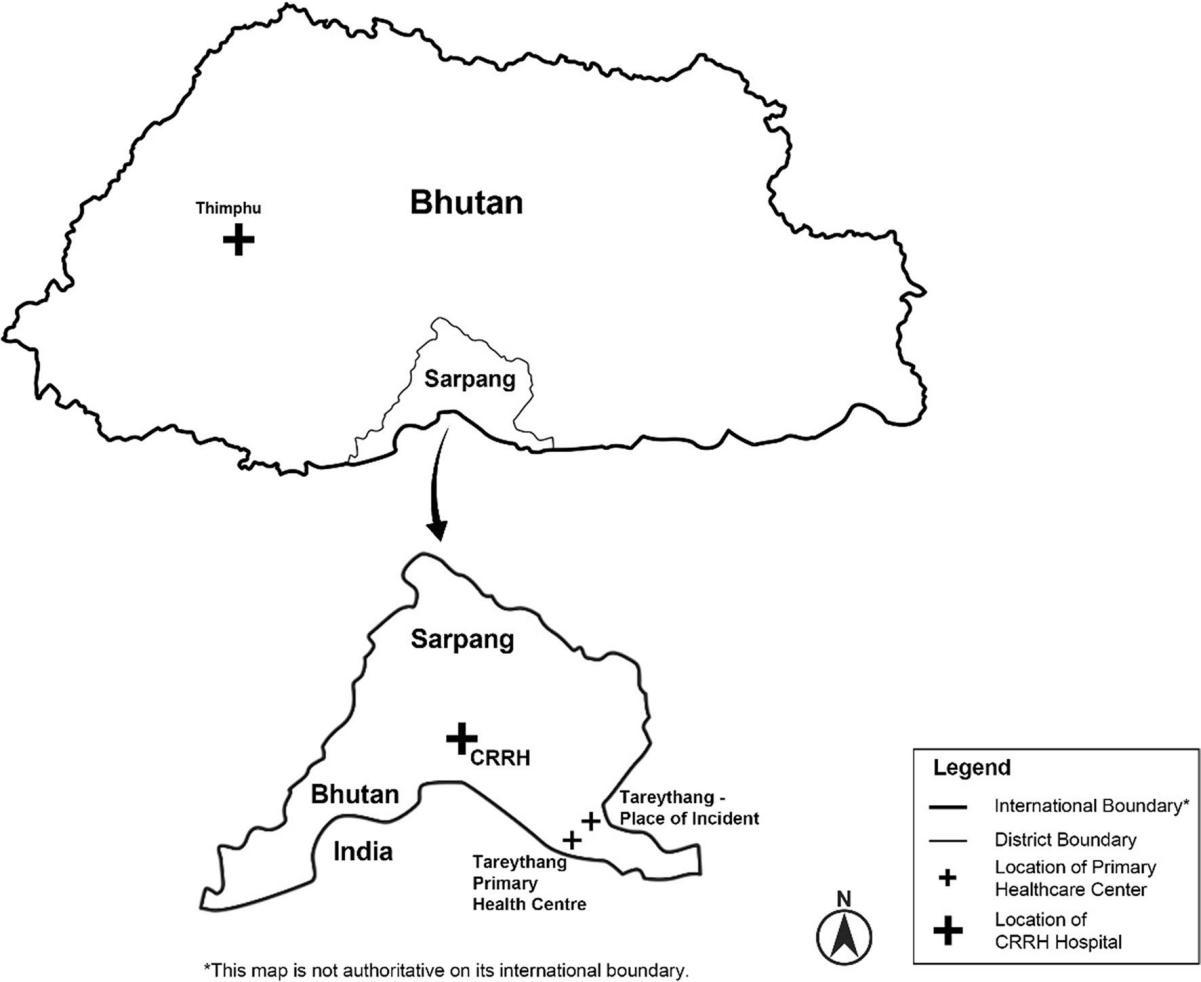
Map of Bhutan showing the border town of Gelephu, the settlement in Tareythang and the Central Regional Referral Hospital (CRRH) where a 35-year-old male for rabies encephalomyelitis was treated in June–July 2023

Twenty-five days prior to admission, the patient had history of dog bite above the right knee resulting in deep laceration on both medial and lateral aspects of the lower thigh. The dog bite happened in Tareythang, a settlement located along the Bhutan-India international border (Fig. 1). The patient had received anti-rabies virus post-exposure prophylaxis, intra-dermal injections (Day 0, 3, and 7) and tetanus toxoid. The dog had bitten four cats (all died due to injuries) and two cows (both died later and one tested positive for rabies using a rapid test kit and fluorescent antibody test on brain sample). The dog was not a native of the place and was chased away, its status unknown.

**Fig. 2:**
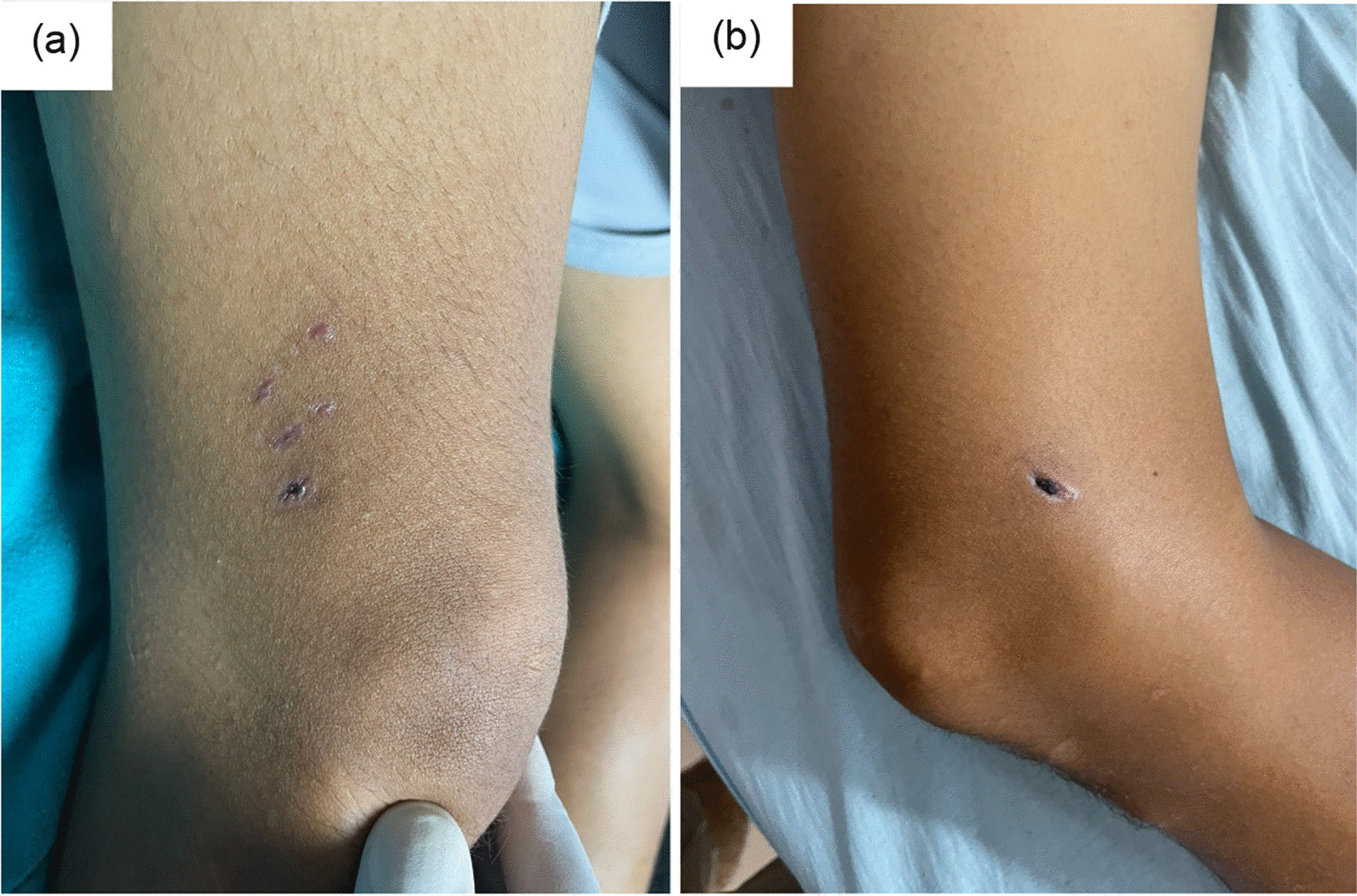
Wounds from a bite from an unknown dog, 25 days prior to admission, in a 35-year-old male with rabies encephalomyelitis. **a** Lateral aspect of lower end of right thigh with three healed lacerations. **b** Medial aspect of right lower end of thigh with one deep puncture wound with healing scab

On initial examination, he was oriented and following instructions, had temperature 38.7 ℃, pulse rate 60–100/min (variable), blood pressure 110/80 mmHg, respiratory rate 22/min, SpO_2_ 98% on room air, random blood glucose 6.9 mmol/L. No pallor, icterus, cyanosis, clubbing, oedema. Goose skin on the anterior aspect of left thigh. He had a bite mark with black healing scar 1 cm on medial side, bite marks with healing lacerations on lateral side of the right thigh, just above the knee joint. The dog bite wound was dry with no evidence of secondary infection (Fig. 2).

Cardiovascular system: S1 S2 normal. Respiratory system: bilateral vesicular breaths. Abdomen: soft, non-tender, no organomegaly, bowel sounds present. Nervous system: higher mental function—oriented to time, place, person. No cranial nerve deficits, no neck stiffness. Aerophobia and hydrophobia present. Spontaneous fasciculations in bilateral thigh and right forearm. Upper limbs: tone normal, bulk normal, power 5 in both proximal and distal muscles. Lower limbs: hypotonia in bilateral lower limbs, power 0 in right lower limb, power 1 in left lower limb. Reflexes: normal in bilateral biceps, triceps and supinator. Bilateral knee joint and ankle joint reflexes absent. Bilateral plantar reflexes flexor. Abdominal reflexes absent below umbilicus. Sensory examination: Hyperasthesia from T_8_ downwards. Touch, vibration (128 Hz), joint position sensation intact.

### Laboratory diagnosis

The findings of the investigations over the course of hospital admission are summarized in Table 1. Polymerase chain reaction for rabies virus in saliva and cerebrospinal fluid tested in Bengaluru, India through the help of the national reference laboratory in Bhutan, were negative. Rabies virus neutralizing antibodies in the cerebrospinal fluid was negative. High titres of rabies virus neutralizing antibodies were found in the serum.

**Table 1:** Summary of investigation findings of a 35-year-old male treated for probable rabies encephalomyelitis at the Central Regional Referral Hospital, Gelephu, Bhutan, 2023

Test parameters	Patient’s value	Normal range
Haemoglobin (g/L)	147	140–180
Mean corpuscular volume (fL)	82	81–99
White blood cell count (/μL)	8690	4000–10000
Neutrophil (%)	92	40–60
Platelet (/mL)	296	150–450
Urea (μmol/L)	3330 13,154	2498–7493
Creatinine (μmol/L)	106 168	53–115
Na/K (mEq/L)	130/3.8 165/5	133–146 / 3.8–5.4
Aspartate/Alanine transaminase (IU/L)	51/32	5–40/5–40
Bilirubin, total/direct (μmol/L)	41/10.3	1.7–20.5/ < 3.5
PT/INR	12.8/0.9	13.6– 7.5/0.8–1.2
Creatine phosphokinase (U/L)	1002	< 171
Lactate dehydrogenase (U/L)	792	105–235
Dengue serology	Negative	
Scrub typhus serology	Negative	
Japanese encephalitis serology	Negative	
Anti-HIV/Anti-HCV/HBsAg	Negative	
Malaria parasite	Not seen	
Blood culture	No growth	
Urine culture	No growth	
Tracheal aspirate culture	No growth	
Electrocardiogram	Normal sinus rhythm	
Cerebrospinal fluid (day 2)	WBC = 50 cells/mm^3^, Neutrophil 20%, lymphocyte 80% Protein/albumin 1.5/1.5 μmol/L No bacteria, no acid-fast bacilli Culture = No growth Rabies virus RT-PCR = Negative	
Cerebrospinal fluid (day 18)	WBC = 250 cells/mm^3^, Neutrophil 15%, lymphocyte 85% Protein/albumin 9.0/7.5 μmol/L No bacteria, no acid-fast bacilli Culture = No growth	
Rapid fluorescent focus inhibition test	Cerebrospinal fluid < 16 (negative) Serum = 2048 (positive)	
Contrast-enhanced computed tomography brain (before admission)	No evidence of intracerebral space occupying lesion, infarct, intracerebral haemorrhage, no meningeal enhancement	
Contrast-enhanced computed tomography brain (day 7)	Hypoattenuation in bilateral basal ganglia and thalamus (suggestive of viral encephalitis) and no evidence of cerebral oedema or brain herniation	
Contrast-enhanced computed tomography brain (day 18)	Progressive global hypoxic ischemic encephalopathy	

### Rabies management

Based on the clinical features and the ongoing rabies virus outbreak in the locality, this case was managed as a case of encephalomyelitis secondary to rabies virus infection. The case was admitted to the Isolation Room in the Intensive Care Unit. Given that this was a Category III bite, Human Rabies Immunoglobulin was given (intramuscular injection 20 units/kg) as local infiltration around the bite site and the patient completed the Anti-rabies Virus vaccine (Day 28) after admission.

The patient had a progressive course with altered sensorium, visual hallucinations, photophobia with restlessness with fasciculations involving the intercostal and neck muscles. He also had several episodes of generalized seizures on the night of admission. The patient had worsening of respiratory function and desaturation for which he was intubated and put on mechanical ventilator. For the initial 3 days, he had Glasgow Coma Scale score 7T with episodes of autonomic dysfunction with palpitations, raised blood pressure and diaphoresis and was sedated with Phenobarbitone. From day 4 onwards, his brain stem reflexes were absent.

Even though evaluation were not suggestive of other aetiologies, the patient received antibacterial (Ceftriaxone 2 g iv q12h and Ampicillin 2 g iv q6h) and antiviral agent (Acyclovir 500 mg iv q8h) for 14 days for empirical coverage. A trial of Ribavirin (1 g q12h via nasogastric tube) was administered from day 12 onwards, but was withheld after 4 days with the development of non-oliguric acute kidney injury.

The patient was managed with supportive nursing care. He had fever with hypotension for 2 days that was followed by refractory shock despite the vasopressors (noradrenaline 250 μg/min iv and adrenaline 160 μg/min iv). He survived three events of cardiac arrests and died after 23 days.

As per the Bhutan National Management Guideline for Rabies 2014, the case was reported to the National Early Warning, Alert Response Surveillance and Information System and the District Livestock Office was alerted. A rapid response team from the district deployed and uncovered a rabies outbreak in three dogs, three cattle, two cats and one rabbit in the locality.

## Discussion

Based on the clinical signs and symptoms in the background of a reliable history of bite from a dog with an unknown vaccination status, the patient was admitted as a probable case of rabies. Evaluation for other aetiologies of encephalitis were negative. For confirmatory testing, samples were transported to the national reference laboratory located in the capital city, Thimphu. However, the laboratory lacked the capability for confirmatory tests and the samples were shipped to Bangalore, India for testing. The availability of confirmatory testing is essential not only for the clinical management of the case but also to inform the public health officials for appropriate outbreak control measures. The WHO recommends a minimum of one reference laboratory in every country with the capacity for rabies diagnosis by currently recommended methods [7].

Sampling for intra-vitam diagnosis remains a major challenge. In a series of eight cases in India, viral RNA by PCR was detected in cerebrospinal fluid in one, in nuchal skin in one and in none in saliva samples [8]. In a patient who has received prior post-exposure prophylaxis (partial or complete), demonstration of at least four-fold rise in the titre of neutralizing antibodies is a means of diagnosis [8]. For post-mortem diagnosis, Negri bodies in brain samples, direct fluorescent antibody tests and antigen detection tests are confirmatory. However, post-mortem testing is not possible most of the times due to socio-cultural sensitivities surrounding handling of bodies after death.

In this case, the victim had received post-exposure prophylaxis with purified vero cell rabies vaccine by intra-dermal route at the Primary Health Care centre as recommended by National Rabies Management Protocol 2014 [9]. Upon investigation by the Ministry of Health, there were no issues with the quality of vaccine, its storage or expiry date. However, as per the National Essential Medicines List 2021, rabies immunoglobulin (equine or human) are available only up to the District Hospitals which are staffed by doctors [10]. As the Primary Health Care centres are staffed by diploma-level Health Assistants, rabies immunoglobulin was not available. While the national protocol describes the indications for immunoglobulins, it misses out a key element on the advice for healthcare workers at the primary level on which types of patients to be referred to the nearest District Hospital.

Rabies is thought of as always fatal with high risk of transmission through saliva of an infected animal. The national protocol specifically disallows referral of a probable case of rabies to higher centres [9]. However, there are a number of rabies survivors albeit with poor neuro-functional status [8, 11]. Agents such as monoclonal antibodies, interferon-α and ribavirin have been suggested having potential activity against RABV [12], but have not been shown useful in clinical reports. Newer antivirals being studied for activities against RABV are favipiravir [13] and galidesvir [14]. In our case, a trial of ribavirin was given as a desperate means to save the patient after discussion with the family members.

The last human case of rabies in Bhutan was that of a 3-year-old child in 2020 that died without access to critical care facility such as intubation and mechanical ventilation [15]. Our case was managed with mechanical ventilation and nursing care that required frequent oral suctioning of saliva. Considering the biosafety aspects of rabies transmission through close contact, personal protective equipment such as gloves, gowns, masks and face shields (leftover from COVID-19 stock) were used, and the healthcare team were provided a full course of post-exposure prophylaxis. However, the national protocol lacks a mention on specific details on the care of rabies in a tertiary care setting and on handling of body after death of such patients.

Bhutan has strong commitment towards controlling rabies and ending human rabies deaths by 2030 [16]. The country has implemented a series of nationwide programmes to catch, neuter and vaccinate the dogs. While the earlier programmes aimed to cover 75% of the dog population [9], the country implemented the National Accelerated Dog Population Management Programme and Rabies Control Programme in September 2021 with a budget of Nu 115 million (US dollar 1.4 million) with an aim to achieve 100% sterilization and vaccination of free roaming dogs by 2023 [16, 17]. By July 2023, more than 61,000 dogs had been sterilized and over 58,000 vaccinated against rabies with an overall 94% coverage [18]. However, rabies control in towns and settlements along the international borders require additional efforts in working with the counterpart local health authorities in the neighbouring Indian towns for effective control. Such cross-border joint control initiatives are implemented for malaria only.

Control of rabies and achieving the 2030 targets requires a One Health approach. Rabies is a nationally notifiable disease that was reported to the Royal Centre for Disease Control through the National Early Warning, Alert Response Surveillance and Information System. After the reporting of this case, rabies immunoglobulins were made available at the Primary Healthcare Centres and Health Assistants were trained on its administration. The hospital developed and implemented a standard operating procedure for handling dead bodies resulting from rabies infection.

The reporting of this human case led to deployment of a Rapid Response Team comprising of human health and livestock officials from the district administration. Some of the outbreak responses included household survey on animal bites that covered 1258 individuals and providing post-exposure prophylaxis to those who had animal bites. Awareness and public advocacy were given to 2782 individuals across 12 sub-districts and a municipality and 8941 students, 571 teachers and 65 school staff across 18 schools. Mass dog vaccination for rabies between June–August 2023 had covered 2229 dogs and 2838 cats. Eighty-three dogs and 55 cats were culled.

## Conclusions

This was a clinically confirmed death resulting from rabies virus infection in the background of rabies outbreak among domestic and wild mammals. The management of this case was resource intensive in critical care setting. To meet the 2030 targets of zero human deaths due to rabies, it is important to provide timely and appropriate guideline-driven treatment to victims of animal bites. For Bhutan, there is an urgent need to update the national rabies management guidelines to address the context-specific gaps. Border towns and settlements along the Bhutan-India border need to implement joint cross-border programmes in the control and prevention of rabies.

## Data Availability

All relevant data sources are cited in the article.

## Abbreviations

PCR: Polymerase chain reaction
RABV: Rabies virus
RNA: Ribonucleic acid
